# Bis{[(η^5^)-cyclopentadienyl]tris(diethyl phosphito-κ^3^ *P*,*P*′,*P*′′)cobaltate(III)-κ^3^ *O*,*O*′,*O*′′]oxovanadium(IV)}-μ-oxalate

**DOI:** 10.1107/S1600536808024458

**Published:** 2008-08-06

**Authors:** Craig C. McLauchlan, Alexander E. Anderson

**Affiliations:** a Department of Chemistry, Illinois State University, Campus Box 4160, Normal, IL 61790-4160, USA

## Abstract

The title compound {systematic name: bis[1,4(η^5^)-cyclo­penta­dien­yl]hexa­kis(μ-diethyl phosphito)-1:2κ^6^ *P*:*O*;3:4κ^6^ *O*:*P*-μ-ox­alato-2:3κ^4^ *O* ^1^,*O* ^2^:*O* ^1′^,*O* ^2′^-dioxido-2κ*O*,3κ*O*-1,4-dicobalt(III)-2,3-divanadium(IV)}, [Co_2_V_2_(C_5_H_5_)_2_(C_2_O_4_)(C_4_H_10_O_3_P)_6_O_2_], is an oxalate-bridged dinuclear complex of oxovanadium(IV). The geometric center of the dimer lies on an inversion center. The unique Co atom is bonded to three P atoms and a cyclo­penta­dienyl ring. The unique V atom has six O atom neighbors in an approximately octa­hedral arrangement; the V—O bond *trans* to the V=O bond is significantly lengthened.

## Related literature

The title compound was synthesized by oxidation of the known {[Cp(P^OEt^)_3_Co]VCl}_2_(μ-C_2_O_4_) dimer (Weberski & McLauchlan, 2007*a* ▶). For related literature on vanadium-oxalate species, see: Salta *et al.* (1996 ▶); Triki *et al.* (2000 ▶); Li *et al.* (2003 ▶); Min *et al.* (2005 ▶); Tatiersky *et al.* (2005 ▶); Yang *et al.* (2006 ▶); Costisor *et al.* (2001 ▶). For related literature on the ligand, see: Kläui (1979 ▶); Kläui *et al.* (1987 ▶); Kamenar *et al.* (1988 ▶); Ward *et al.* (1998 ▶); Kölle & Englert (2002 ▶); Weberski & McLauchlan (2007**b* ▶)*.
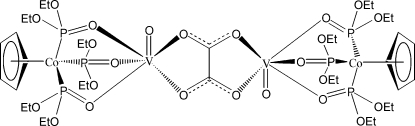


## Experimental

### 

#### Crystal data

[Co_2_V_2_(C_5_H_5_)_2_(C_2_O_4_)(C_4_H_10_O_3_P)_6_O_2_]
*M* *_r_* = 1292.48
Monoclinic, 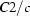

*a* = 28.364 (5) Å
*b* = 10.9825 (18) Å
*c* = 19.976 (3) Å
β = 117.412 (2)°
*V* = 5524.0 (16) Å^3^
*Z* = 4
Mo *K*α radiation
μ = 1.17 mm^−1^
*T* = 180 (2) K
0.63 × 0.33 × 0.06 mm

#### Data collection

Bruker SMART APEX CCD diffractometer
Absorption correction: multi-scan (*APEX2*; Bruker, 2008 ▶) *T*_min_ = 0.682, *T*_max_ = 0.932
26145 measured reflections
6845 independent reflections
5617 reflections with *I* > 2σ(*I*)
*R*_int_ = 0.036

#### Refinement

*R*[*F* ^2^ > 2σ(*F* ^2^)] = 0.040
*wR*(*F* ^2^) = 0.109
*S* = 1.00
6845 reflections
323 parameters
H-atom parameters constrained
Δρ_max_ = 0.70 e Å^−3^
Δρ_min_ = −0.45 e Å^−3^

### 

Data collection: *APEX2* (Bruker, 2008 ▶); cell refinement: *APEX2*; data reduction: *APEX2*; program(s) used to solve structure: *SHELXTL* (Sheldrick, 2008 ▶); program(s) used to refine structure: *SHELXTL*; molecular graphics: *SHELXTL*; software used to prepare material for publication: *SHELXTL*.

## Supplementary Material

Crystal structure: contains datablocks I, global. DOI: 10.1107/S1600536808024458/ww2126sup1.cif

Structure factors: contains datablocks I. DOI: 10.1107/S1600536808024458/ww2126Isup2.hkl

Additional supplementary materials:  crystallographic information; 3D view; checkCIF report

## Figures and Tables

**Table:** 

V1—O3	1.594 (2)
V1—O1*P*	1.9932 (18)
V1—O3*P*	2.0145 (18)
V1—O1	2.0490 (18)
V1—O2	2.0727 (17)
V1—O2*P*	2.2077 (18)

**Table:** 

O3—V1—O1*P*	97.46 (9)
O3—V1—O3*P*	99.73 (9)
O1*P*—V1—O3*P*	91.75 (7)
O3—V1—O1	99.26 (9)
O1*P*—V1—O1	89.59 (7)
O3*P*—V1—O1	160.61 (8)
O3—V1—O2	96.64 (9)
O1*P*—V1—O2	164.10 (8)
O3*P*—V1—O2	93.07 (7)
O1—V1—O2	80.91 (7)
O3—V1—O2*P*	177.13 (9)
O1*P*—V1—O2*P*	84.59 (7)
O3*P*—V1—O2*P*	82.17 (7)
O1—V1—O2*P*	78.71 (7)
O2—V1—O2*P*	81.07 (7)
C1—O1—V1	112.73 (16)
C2—O2—V1	112.22 (17)

Symmetry code: (i) 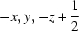.

**Fig. 1.**
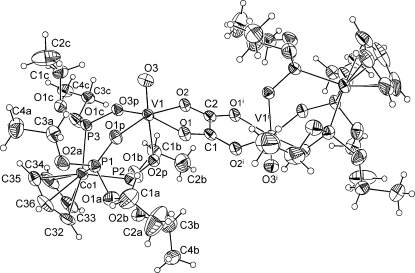
View of the molecular structure of (I) showing 50% displacement ellipsoids (arbitrary spheres for the H atoms). Symmetry code: (i) -x, y, -z + 1/2.

## supplementary crystallographic information

### Comment

The title compound, {[Cp(P^OEt^)_3_Co]VO}_2_(µ-C_2_O_4_), (I), (Cp = cyclopentadienyl anion, C_5_H_5_^-^, P^OEt^ = diethylphosphite anion, C_4_H_10_O_3_P^-^) was synthesized by oxidation of the known {[Cp(P^OEt^)_3_Co]VCl}_2_(µ-C_2_O_4_) dimer (Weberski & McLauchlan, 2007*a*).

Dozens of both terminal (*e.g.*, Tatiersky *et al.*, 2005; Costisor *et al.*, 2006) and bridging (*e.g.*, Salta *et al.*, 1996; Triki *et al.*, 2000; Li *et al.*, 2003; Min *et al.*, 2005; Yang *et al.*, 2006) oxalato complexes have been reported for vanadium. The distances and angles in (I) (Table 1) are comparable to those in these known reported structures for oxalate bridging complexes. Similarly, the distances and angles in (I) are comparable to the previously reported structures involving the ligand (Kläui, 1979; Kläui *et al.*, 1987; Kamenar *et al.*, 1988; Ward *et al.*, 1998; Kölle & Englert, 2002; Weberski & McLauchlan, 2007*b*)

The geometric center of the dimer lies on the inversion center (Fig. 1).

Some minor disorder, as may be expected, is present in the ethyl groups and the Cp ring, which results in some slightly elongated ellipsoids, but the disorder was not modeled.

### Experimental

Compound (I) was synthesized by serendipitous air oxidation of the known {[Cp(P^OEt^)_3_Co]VCl}_2_(µ-C_2_O_4_) dimer (Weberski & McLauchlan, 2007*a*) in diethyl ether (Cp = cyclopentadienyl anion, C_5_H_5_^-^, P^OEt^ = diethylphosphite anion, C_4_H_10_O_3_P^-^). An amount of 44 mg of green, X-ray diffraction quality crystals of (I) were grown from the unoptimized slow evaporation of an ether solution at *ca* 300 K. The crystals shatter at 100 K. Anal. Calcd. for C_36_H_70_Co_2_O_24_P_6_V_2_: C, 33.45; H, 5.46. Found: C, 33.85; H, 5.65. The compound decomposes above 473 K. Infrared (cm^-1^): 441, 478, 590, 728, 771, 835, 930, 977, 1031, 1129, 1262, 1353, 1388, 1426, 1444, 1477, 1625, 2345, 2367, 2930, 2979, 3423. Magnetic susceptibility (Evans method, uncorrected), χ_m_ (χ_m_T) 1.65 *x* 10^-3^ erg*G^-2^mol^-1^ (0.492). Electronic absorbance (UV/vis, CH_3_CN, λ, nm(ε, *M*^-1^ cm^-1^)): 242 (42000), 333 (5700), 485 (124), 656 (116).

### Refinement

The H atoms were geometrically placed (C—H = 0.95–0.99 Å) and refined as riding with *U*_iso_(H) = 1.2*U*_iso_(C) or 1.5*U*_eq_(methyl C).

### Crystal data

**Table:**

[Co_2_V_2_(C_5_H_5_)_2_(C_2_O_4_)(C_4_H_10_O_3_P)_6_O_2_]	*F*(000) = 2672
*M**_r_* = 1292.48	*D*_x_ = 1.554 Mg m^−^^3^
Monoclinic, *C*2/*c*	Melting point: dec. 473(1) K
Hall symbol: -C 2yc	Mo *K*α radiation, λ = 0.71073 Å
*a* = 28.364 (5) Å	Cell parameters from 9976 reflections
*b* = 10.9825 (18) Å	θ = 5.0–63.5°
*c* = 19.976 (3) Å	µ = 1.17 mm^−^^1^
β = 117.412 (2)°	*T* = 180 K
*V* = 5524.0 (16) Å^3^	Plate, green
*Z* = 4	0.63 × 0.33 × 0.06 mm

### Data collection

**Table:**

Bruker SMART APEX CCD diffractometer	6845 independent reflections
Radiation source: fine-focus sealed tube	5617 reflections with *I* > 2σ(*I*)
graphite	*R*_int_ = 0.037
ω scans	θ_max_ = 28.3°, θ_min_ = 2.0°
Absorption correction: multi-scan (*APEX2*; Bruker, 2008)	*h* = −37→37
*T*_min_ = 0.682, *T*_max_ = 0.932	*k* = −14→14
26145 measured reflections	*l* = −26→26

### Refinement

**Table:**

Refinement on *F*^2^	Primary atom site location: structure-invariant direct methods
Least-squares matrix: full	Secondary atom site location: difference Fourier map
*R*[*F*^2^ > 2σ(*F*^2^)] = 0.040	Hydrogen site location: inferred from neighbouring sites
*wR*(*F*^2^) = 0.109	H-atom parameters constrained
*S* = 1.00	*w* = 1/[σ^2^(*F*_o_^2^) + (0.0479*P*)^2^ + 16.0571*P*] where *P* = (*F*_o_^2^ + 2*F*_c_^2^)/3
6845 reflections	(Δ/σ)_max_ = 0.001
323 parameters	Δρ_max_ = 0.70 e Å^−^^3^
0 restraints	Δρ_min_ = −0.45 e Å^−^^3^

### Special details

**Table:**

Geometry. All e.s.d.'s (except the e.s.d. in the dihedral angle between two l.s. planes) are estimated using the full covariance matrix. The cell e.s.d.'s are taken into account individually in the estimation of e.s.d.'s in distances, angles and torsion angles; correlations between e.s.d.'s in cell parameters are only used when they are defined by crystal symmetry. An approximate (isotropic) treatment of cell e.s.d.'s is used for estimating e.s.d.'s involving l.s. planes.
Refinement. Refinement of *F*^2^ against ALL reflections. The weighted *R*-factor *wR* and goodness of fit *S* are based on *F*^2^, conventional *R*-factors *R* are based on *F*, with *F* set to zero for negative *F*^2^. The threshold expression of *F*^2^ > σ(*F*^2^) is used only for calculating *R*-factors(gt) *etc*. and is not relevant to the choice of reflections for refinement. *R*-factors based on *F*^2^ are statistically about twice as large as those based on *F*, and *R*- factors based on ALL data will be even larger.

### Fractional atomic coordinates and isotropic or equivalent isotropic displacement parameters (Å^2^)

**Table:**

	*x*	*y*	*z*	*U*_iso_*/*U*_eq_	
V1	0.028671 (16)	0.78146 (4)	0.13941 (2)	0.02455 (10)	
Co1	0.176050 (13)	0.74072 (3)	0.16646 (2)	0.02774 (10)	
P1	0.11499 (3)	0.60266 (6)	0.12884 (4)	0.02764 (14)	
P2	0.15939 (3)	0.78092 (6)	0.26021 (4)	0.02754 (14)	
P3	0.11777 (3)	0.87517 (6)	0.09797 (4)	0.02816 (15)	
O1	0.00650 (7)	0.66231 (15)	0.19874 (10)	0.0286 (4)	
O2	0.01090 (7)	0.90562 (15)	0.20266 (10)	0.0275 (4)	
C1	0.0000	0.7134 (3)	0.2500	0.0245 (7)	
C2	0.0000	0.8544 (3)	0.2500	0.0248 (7)	
O3	−0.02642 (8)	0.79215 (18)	0.06448 (11)	0.0379 (4)	
O1P	0.05862 (7)	0.64296 (16)	0.10672 (11)	0.0309 (4)	
O1A	0.13253 (8)	0.50150 (17)	0.19333 (11)	0.0375 (4)	
O2A	0.11265 (8)	0.5267 (2)	0.05926 (12)	0.0428 (5)	
C1A	0.09433 (16)	0.4095 (3)	0.1891 (2)	0.0563 (9)	
H1AA	0.0580	0.4430	0.1613	0.068*	
H1AB	0.0970	0.3382	0.1606	0.068*	
C2A	0.1032 (2)	0.3716 (6)	0.2616 (3)	0.105 (2)	
H2AA	0.0772	0.3089	0.2567	0.157*	
H2AB	0.0994	0.4415	0.2893	0.157*	
H2AC	0.1392	0.3383	0.2892	0.157*	
C3A	0.06647 (12)	0.5172 (3)	−0.01328 (17)	0.0377 (6)	
H3AA	0.0420	0.5859	−0.0204	0.045*	
H3AB	0.0474	0.4402	−0.0163	0.045*	
C4A	0.08407 (16)	0.5198 (4)	−0.0731 (2)	0.0567 (9)	
H4AA	0.0530	0.5145	−0.1228	0.085*	
H4AB	0.1077	0.4506	−0.0663	0.085*	
H4AC	0.1031	0.5960	−0.0695	0.085*	
O2P	0.10274 (7)	0.76760 (15)	0.24648 (10)	0.0273 (4)	
O1B	0.18059 (8)	0.91352 (19)	0.29463 (14)	0.0444 (5)	
O2B	0.19748 (8)	0.6950 (2)	0.32940 (11)	0.0397 (5)	
C1B	0.14588 (12)	1.0097 (3)	0.29536 (18)	0.0403 (6)	
H1BA	0.1091	0.9928	0.2566	0.048*	
H1BB	0.1570	1.0881	0.2826	0.048*	
C2B	0.1473 (2)	1.0196 (5)	0.3698 (3)	0.0848 (15)	
H2BA	0.1262	1.0900	0.3702	0.127*	
H2BB	0.1841	1.0301	0.4087	0.127*	
H2BC	0.1325	0.9454	0.3800	0.127*	
C3B	0.18998 (16)	0.6875 (4)	0.3955 (2)	0.0542 (9)	
H3BA	0.1516	0.6810	0.3806	0.065*	
H3BB	0.2038	0.7621	0.4261	0.065*	
C4B	0.21855 (14)	0.5785 (3)	0.4410 (2)	0.0535 (8)	
H4BA	0.2162	0.5779	0.4884	0.080*	
H4BB	0.2559	0.5818	0.4519	0.080*	
H4BC	0.2022	0.5043	0.4124	0.080*	
O3P	0.07192 (7)	0.90314 (15)	0.11549 (10)	0.0287 (4)	
O1C	0.09368 (8)	0.83591 (19)	0.01116 (11)	0.0396 (5)	
O2C	0.14763 (8)	1.00128 (18)	0.10399 (14)	0.0469 (6)	
C1C	0.04967 (13)	0.9052 (3)	−0.04630 (17)	0.0448 (7)	
H1CA	0.0637	0.9746	−0.0633	0.054*	
H1CB	0.0270	0.9378	−0.0248	0.054*	
C2C	0.01791 (18)	0.8257 (3)	−0.1108 (2)	0.0791 (15)	
H2CA	−0.0109	0.8730	−0.1500	0.119*	
H2CB	0.0028	0.7590	−0.0942	0.119*	
H2CC	0.0406	0.7921	−0.1312	0.119*	
C3C	0.12391 (11)	1.1198 (2)	0.09617 (17)	0.0337 (6)	
H3CA	0.1295	1.1500	0.1460	0.040*	
H3CB	0.0852	1.1152	0.0624	0.040*	
C4C	0.14959 (12)	1.2044 (3)	0.0636 (2)	0.0435 (7)	
H4CA	0.1356	1.2869	0.0607	0.065*	
H4CB	0.1419	1.1767	0.0129	0.065*	
H4CC	0.1881	1.2048	0.0959	0.065*	
C32	0.24880 (11)	0.6532 (3)	0.22456 (19)	0.0429 (7)	
H32	0.2576	0.5990	0.2656	0.051*	
C33	0.25670 (12)	0.7796 (3)	0.2303 (2)	0.0523 (9)	
H33	0.2718	0.8255	0.2757	0.063*	
C34	0.23856 (15)	0.8257 (4)	0.1582 (3)	0.0677 (13)	
H34	0.2394	0.9088	0.1455	0.081*	
C35	0.21853 (15)	0.7274 (5)	0.1062 (2)	0.0696 (13)	
H35	0.2030	0.7329	0.0529	0.084*	
C36	0.22599 (12)	0.6204 (3)	0.1488 (2)	0.0512 (8)	
H36	0.2170	0.5401	0.1293	0.061*	

### Atomic displacement parameters (Å^2^)

**Table:**

	*U*^11^	*U*^22^	*U*^33^	*U*^12^	*U*^13^	*U*^23^
V1	0.0245 (2)	0.02041 (19)	0.0309 (2)	0.00117 (15)	0.01458 (17)	−0.00023 (15)
Co1	0.02674 (18)	0.02207 (16)	0.0390 (2)	0.00463 (12)	0.01910 (15)	0.00510 (13)
P1	0.0314 (3)	0.0205 (3)	0.0336 (3)	0.0033 (2)	0.0171 (3)	−0.0002 (2)
P2	0.0246 (3)	0.0253 (3)	0.0329 (3)	−0.0018 (2)	0.0135 (3)	−0.0004 (2)
P3	0.0298 (3)	0.0221 (3)	0.0388 (4)	0.0052 (2)	0.0212 (3)	0.0071 (3)
O1	0.0325 (9)	0.0175 (8)	0.0429 (10)	−0.0020 (6)	0.0236 (8)	−0.0026 (7)
O2	0.0315 (9)	0.0180 (7)	0.0401 (10)	0.0001 (6)	0.0225 (8)	0.0006 (7)
C1	0.0187 (14)	0.0185 (14)	0.0369 (18)	0.000	0.0133 (13)	0.000
C2	0.0228 (15)	0.0172 (14)	0.0347 (18)	0.000	0.0135 (14)	0.000
O3	0.0314 (10)	0.0348 (10)	0.0419 (11)	0.0034 (8)	0.0120 (9)	0.0001 (8)
O1P	0.0326 (9)	0.0253 (8)	0.0389 (10)	−0.0003 (7)	0.0198 (8)	−0.0065 (7)
O1A	0.0433 (11)	0.0241 (9)	0.0435 (11)	−0.0020 (8)	0.0185 (9)	0.0064 (8)
O2A	0.0421 (11)	0.0443 (12)	0.0396 (11)	0.0125 (9)	0.0168 (9)	−0.0094 (9)
C1A	0.072 (2)	0.0375 (17)	0.058 (2)	−0.0188 (16)	0.0285 (19)	0.0034 (15)
C2A	0.118 (4)	0.133 (5)	0.067 (3)	−0.067 (4)	0.046 (3)	0.004 (3)
C3A	0.0390 (15)	0.0340 (14)	0.0414 (15)	−0.0036 (11)	0.0196 (12)	−0.0083 (12)
C4A	0.063 (2)	0.068 (2)	0.0461 (19)	−0.0039 (18)	0.0307 (17)	−0.0128 (17)
O2P	0.0272 (9)	0.0254 (8)	0.0306 (9)	−0.0009 (7)	0.0144 (7)	0.0002 (7)
O1B	0.0359 (11)	0.0347 (10)	0.0677 (14)	−0.0093 (8)	0.0282 (10)	−0.0180 (10)
O2B	0.0324 (10)	0.0495 (12)	0.0351 (10)	0.0043 (9)	0.0138 (8)	0.0074 (9)
C1B	0.0377 (15)	0.0304 (13)	0.0549 (18)	−0.0032 (11)	0.0232 (14)	−0.0044 (12)
C2B	0.113 (4)	0.084 (3)	0.079 (3)	0.037 (3)	0.063 (3)	0.007 (3)
C3B	0.066 (2)	0.054 (2)	0.0445 (18)	0.0085 (17)	0.0268 (17)	0.0085 (16)
C4B	0.0481 (19)	0.061 (2)	0.0481 (19)	0.0047 (16)	0.0192 (15)	0.0151 (16)
O3P	0.0307 (9)	0.0229 (8)	0.0380 (10)	0.0055 (7)	0.0205 (8)	0.0054 (7)
O1C	0.0442 (11)	0.0435 (11)	0.0368 (11)	0.0169 (9)	0.0234 (9)	0.0103 (9)
O2C	0.0412 (11)	0.0224 (9)	0.0897 (18)	0.0063 (8)	0.0407 (12)	0.0144 (10)
C1C	0.0518 (18)	0.0427 (16)	0.0379 (16)	0.0138 (14)	0.0190 (14)	0.0120 (13)
C2C	0.076 (3)	0.0360 (18)	0.077 (3)	−0.0013 (18)	−0.006 (2)	0.0073 (18)
C3C	0.0346 (13)	0.0231 (12)	0.0444 (15)	0.0049 (10)	0.0191 (12)	0.0044 (11)
C4C	0.0403 (16)	0.0289 (13)	0.0593 (19)	0.0005 (12)	0.0212 (14)	0.0141 (13)
C32	0.0280 (13)	0.0427 (16)	0.0587 (19)	0.0143 (12)	0.0206 (13)	0.0114 (14)
C33	0.0287 (14)	0.0448 (17)	0.085 (3)	−0.0015 (13)	0.0273 (16)	−0.0089 (17)
C34	0.047 (2)	0.050 (2)	0.132 (4)	0.0157 (16)	0.063 (2)	0.034 (2)
C35	0.046 (2)	0.122 (4)	0.057 (2)	0.035 (2)	0.0371 (18)	0.028 (2)
C36	0.0349 (16)	0.0471 (18)	0.076 (2)	0.0107 (13)	0.0294 (16)	−0.0104 (17)

### Geometric parameters (Å, °)

**Table:**

V1—O3	1.594 (2)	C4A—H4AB	0.9800
V1—O1P	1.9932 (18)	C4A—H4AC	0.9800
V1—O3P	2.0145 (18)	O1B—C1B	1.449 (3)
V1—O1	2.0490 (18)	O2B—C3B	1.433 (4)
V1—O2	2.0727 (17)	C1B—C2B	1.473 (5)
V1—O2P	2.2077 (18)	C1B—H1BA	0.9900
Co1—C35	2.063 (3)	C1B—H1BB	0.9900
Co1—C34	2.077 (3)	C2B—H2BA	0.9800
Co1—C32	2.079 (3)	C2B—H2BB	0.9800
Co1—C36	2.082 (3)	C2B—H2BC	0.9800
Co1—C33	2.087 (3)	C3B—C4B	1.496 (5)
Co1—P1	2.1597 (8)	C3B—H3BA	0.9900
Co1—P3	2.1684 (7)	C3B—H3BB	0.9900
Co1—P2	2.1774 (8)	C4B—H4BA	0.9800
P1—O1P	1.5156 (19)	C4B—H4BB	0.9800
P1—O2A	1.596 (2)	C4B—H4BC	0.9800
P1—O1A	1.597 (2)	O1C—C1C	1.461 (3)
P2—O2P	1.5075 (18)	O2C—C3C	1.441 (3)
P2—O1B	1.604 (2)	C1C—C2C	1.471 (5)
P2—O2B	1.610 (2)	C1C—H1CA	0.9900
P3—O3P	1.5257 (18)	C1C—H1CB	0.9900
P3—O2C	1.599 (2)	C2C—H2CA	0.9800
P3—O1C	1.603 (2)	C2C—H2CB	0.9800
O1—C1	1.253 (2)	C2C—H2CC	0.9800
O2—C2	1.255 (2)	C3C—C4C	1.501 (4)
C1—O1^i^	1.254 (2)	C3C—H3CA	0.9900
C1—C2	1.549 (4)	C3C—H3CB	0.9900
C2—O2^i^	1.255 (2)	C4C—H4CA	0.9800
O1A—C1A	1.455 (4)	C4C—H4CB	0.9800
O2A—C3A	1.442 (3)	C4C—H4CC	0.9800
C1A—C2A	1.415 (5)	C32—C36	1.391 (5)
C1A—H1AA	0.9900	C32—C33	1.402 (5)
C1A—H1AB	0.9900	C32—H32	0.9500
C2A—H2AA	0.9800	C33—C34	1.383 (6)
C2A—H2AB	0.9800	C33—H33	0.9500
C2A—H2AC	0.9800	C34—C35	1.422 (7)
C3A—C4A	1.492 (4)	C34—H34	0.9500
C3A—H3AA	0.9900	C35—C36	1.409 (6)
C3A—H3AB	0.9900	C35—H35	0.9500
C4A—H4AA	0.9800	C36—H36	0.9500
			
O3—V1—O1P	97.46 (9)	C3A—C4A—H4AA	109.5
O3—V1—O3P	99.73 (9)	C3A—C4A—H4AB	109.5
O1P—V1—O3P	91.75 (7)	H4AA—C4A—H4AB	109.5
O3—V1—O1	99.26 (9)	C3A—C4A—H4AC	109.5
O1P—V1—O1	89.59 (7)	H4AA—C4A—H4AC	109.5
O3P—V1—O1	160.61 (8)	H4AB—C4A—H4AC	109.5
O3—V1—O2	96.64 (9)	P2—O2P—V1	128.96 (11)
O1P—V1—O2	164.10 (8)	C1B—O1B—P2	122.94 (18)
O3P—V1—O2	93.07 (7)	C3B—O2B—P2	119.9 (2)
O1—V1—O2	80.91 (7)	O1B—C1B—C2B	111.2 (3)
O3—V1—O2P	177.13 (9)	O1B—C1B—H1BA	109.4
O1P—V1—O2P	84.59 (7)	C2B—C1B—H1BA	109.4
O3P—V1—O2P	82.17 (7)	O1B—C1B—H1BB	109.4
O1—V1—O2P	78.71 (7)	C2B—C1B—H1BB	109.4
O2—V1—O2P	81.07 (7)	H1BA—C1B—H1BB	108.0
C35—Co1—C34	40.17 (18)	C1B—C2B—H2BA	109.5
C35—Co1—C32	66.22 (14)	C1B—C2B—H2BB	109.5
C34—Co1—C32	65.66 (14)	H2BA—C2B—H2BB	109.5
C35—Co1—C36	39.74 (16)	C1B—C2B—H2BC	109.5
C34—Co1—C36	66.39 (15)	H2BA—C2B—H2BC	109.5
C32—Co1—C36	39.06 (14)	H2BB—C2B—H2BC	109.5
C35—Co1—C33	66.45 (17)	O2B—C3B—C4B	109.7 (3)
C34—Co1—C33	38.81 (17)	O2B—C3B—H3BA	109.7
C32—Co1—C33	39.33 (13)	C4B—C3B—H3BA	109.7
C36—Co1—C33	66.01 (14)	O2B—C3B—H3BB	109.7
C35—Co1—P1	109.73 (16)	C4B—C3B—H3BB	109.7
C34—Co1—P1	149.89 (15)	H3BA—C3B—H3BB	108.2
C32—Co1—P1	107.49 (9)	C3B—C4B—H4BA	109.5
C36—Co1—P1	89.69 (10)	C3B—C4B—H4BB	109.5
C33—Co1—P1	146.52 (10)	H4BA—C4B—H4BB	109.5
C35—Co1—P3	99.24 (11)	C3B—C4B—H4BC	109.5
C34—Co1—P3	94.27 (10)	H4BA—C4B—H4BC	109.5
C32—Co1—P3	159.92 (9)	H4BB—C4B—H4BC	109.5
C36—Co1—P3	134.90 (11)	P3—O3P—V1	126.68 (10)
C33—Co1—P3	123.29 (10)	C1C—O1C—P3	119.63 (19)
P1—Co1—P3	90.13 (3)	C3C—O2C—P3	124.74 (17)
C35—Co1—P2	158.80 (14)	O1C—C1C—C2C	109.7 (3)
C34—Co1—P2	120.59 (15)	O1C—C1C—H1CA	109.7
C32—Co1—P2	99.57 (10)	C2C—C1C—H1CA	109.7
C36—Co1—P2	135.08 (11)	O1C—C1C—H1CB	109.7
C33—Co1—P2	92.52 (11)	C2C—C1C—H1CB	109.7
P1—Co1—P2	89.13 (3)	H1CA—C1C—H1CB	108.2
P3—Co1—P2	90.02 (3)	C1C—C2C—H2CA	109.5
O1P—P1—O2A	106.46 (11)	C1C—C2C—H2CB	109.5
O1P—P1—O1A	109.47 (11)	H2CA—C2C—H2CB	109.5
O2A—P1—O1A	102.22 (12)	C1C—C2C—H2CC	109.5
O1P—P1—Co1	117.76 (8)	H2CA—C2C—H2CC	109.5
O2A—P1—Co1	112.03 (9)	H2CB—C2C—H2CC	109.5
O1A—P1—Co1	107.79 (8)	O2C—C3C—C4C	108.1 (2)
O2P—P2—O1B	108.90 (10)	O2C—C3C—H3CA	110.1
O2P—P2—O2B	109.71 (11)	C4C—C3C—H3CA	110.1
O1B—P2—O2B	101.13 (12)	O2C—C3C—H3CB	110.1
O2P—P2—Co1	117.32 (8)	C4C—C3C—H3CB	110.1
O1B—P2—Co1	111.45 (9)	H3CA—C3C—H3CB	108.4
O2B—P2—Co1	107.08 (8)	C3C—C4C—H4CA	109.5
O3P—P3—O2C	106.64 (10)	C3C—C4C—H4CB	109.5
O3P—P3—O1C	108.57 (11)	H4CA—C4C—H4CB	109.5
O2C—P3—O1C	105.17 (13)	C3C—C4C—H4CC	109.5
O3P—P3—Co1	118.37 (7)	H4CA—C4C—H4CC	109.5
O2C—P3—Co1	108.60 (8)	H4CB—C4C—H4CC	109.5
O1C—P3—Co1	108.72 (8)	C36—C32—C33	108.8 (3)
C1—O1—V1	112.73 (16)	C36—C32—Co1	70.59 (17)
C2—O2—V1	112.22 (17)	C33—C32—Co1	70.64 (17)
O1—C1—O1^i^	126.8 (3)	C36—C32—H32	125.6
O1—C1—C2	116.59 (15)	C33—C32—H32	125.6
O1^i^—C1—C2	116.59 (15)	Co1—C32—H32	124.8
O2—C2—O2^i^	126.8 (3)	C34—C33—C32	108.0 (4)
O2—C2—C1	116.60 (15)	C34—C33—Co1	70.2 (2)
O2^i^—C2—C1	116.60 (15)	C32—C33—Co1	70.03 (17)
P1—O1P—V1	132.76 (11)	C34—C33—H33	126.0
C1A—O1A—P1	119.3 (2)	C32—C33—H33	126.0
C3A—O2A—P1	124.59 (18)	Co1—C33—H33	125.3
C2A—C1A—O1A	111.6 (3)	C33—C34—C35	108.3 (3)
C2A—C1A—H1AA	109.3	C33—C34—Co1	70.99 (19)
O1A—C1A—H1AA	109.3	C35—C34—Co1	69.4 (2)
C2A—C1A—H1AB	109.3	C33—C34—H34	125.8
O1A—C1A—H1AB	109.3	C35—C34—H34	125.8
H1AA—C1A—H1AB	108.0	Co1—C34—H34	125.4
C1A—C2A—H2AA	109.5	C36—C35—C34	107.1 (3)
C1A—C2A—H2AB	109.5	C36—C35—Co1	70.88 (19)
H2AA—C2A—H2AB	109.5	C34—C35—Co1	70.5 (2)
C1A—C2A—H2AC	109.5	C36—C35—H35	126.4
H2AA—C2A—H2AC	109.5	C34—C35—H35	126.4
H2AB—C2A—H2AC	109.5	Co1—C35—H35	123.9
O2A—C3A—C4A	108.6 (3)	C32—C36—C35	107.8 (3)
O2A—C3A—H3AA	110.0	C32—C36—Co1	70.35 (17)
C4A—C3A—H3AA	110.0	C35—C36—Co1	69.38 (19)
O2A—C3A—H3AB	110.0	C32—C36—H36	126.1
C4A—C3A—H3AB	110.0	C35—C36—H36	126.1
H3AA—C3A—H3AB	108.3	Co1—C36—H36	125.7
			
C35—Co1—P1—O1P	−128.14 (15)	O3P—V1—O2P—P2	−32.47 (13)
C34—Co1—P1—O1P	−127.0 (2)	O1—V1—O2P—P2	150.71 (14)
C32—Co1—P1—O1P	161.50 (13)	O2—V1—O2P—P2	−126.85 (14)
C36—Co1—P1—O1P	−163.17 (14)	O2P—P2—O1B—C1B	−12.3 (3)
C33—Co1—P1—O1P	155.0 (2)	O2B—P2—O1B—C1B	−127.8 (2)
P3—Co1—P1—O1P	−28.28 (9)	Co1—P2—O1B—C1B	118.7 (2)
P2—Co1—P1—O1P	61.74 (9)	O2P—P2—O2B—C3B	−42.2 (3)
C35—Co1—P1—O2A	−4.20 (15)	O1B—P2—O2B—C3B	72.7 (3)
C34—Co1—P1—O2A	−3.1 (2)	Co1—P2—O2B—C3B	−170.6 (2)
C32—Co1—P1—O2A	−74.56 (13)	P2—O1B—C1B—C2B	99.8 (4)
C36—Co1—P1—O2A	−39.23 (14)	P2—O2B—C3B—C4B	163.9 (2)
C33—Co1—P1—O2A	−81.1 (2)	O2C—P3—O3P—V1	−165.84 (14)
P3—Co1—P1—O2A	95.66 (9)	O1C—P3—O3P—V1	81.30 (15)
P2—Co1—P1—O2A	−174.32 (9)	Co1—P3—O3P—V1	−43.18 (16)
C35—Co1—P1—O1A	107.49 (14)	O3—V1—O3P—P3	−109.89 (15)
C34—Co1—P1—O1A	108.6 (2)	O1P—V1—O3P—P3	−12.01 (14)
C32—Co1—P1—O1A	37.13 (13)	O1—V1—O3P—P3	81.7 (3)
C36—Co1—P1—O1A	72.46 (14)	O2—V1—O3P—P3	152.83 (14)
C33—Co1—P1—O1A	30.6 (2)	O2P—V1—O3P—P3	72.28 (14)
P3—Co1—P1—O1A	−152.64 (9)	O3P—P3—O1C—C1C	44.0 (2)
P2—Co1—P1—O1A	−62.63 (9)	O2C—P3—O1C—C1C	−69.8 (2)
C35—Co1—P2—O2P	173.1 (4)	Co1—P3—O1C—C1C	174.1 (2)
C34—Co1—P2—O2P	151.66 (14)	O3P—P3—O2C—C3C	−22.9 (3)
C32—Co1—P2—O2P	−141.04 (12)	O1C—P3—O2C—C3C	92.3 (3)
C36—Co1—P2—O2P	−122.13 (16)	Co1—P3—O2C—C3C	−151.5 (2)
C33—Co1—P2—O2P	−179.98 (13)	P3—O1C—C1C—C2C	−152.3 (3)
P1—Co1—P2—O2P	−33.44 (8)	P3—O2C—C3C—C4C	−149.4 (2)
P3—Co1—P2—O2P	56.69 (8)	C35—Co1—C32—C36	−37.8 (2)
C35—Co1—P2—O1B	46.6 (4)	C34—Co1—C32—C36	−81.9 (3)
C34—Co1—P2—O1B	25.17 (15)	C33—Co1—C32—C36	−119.1 (3)
C32—Co1—P2—O1B	92.47 (13)	P1—Co1—C32—C36	66.6 (2)
C36—Co1—P2—O1B	111.37 (17)	P3—Co1—C32—C36	−83.8 (3)
C33—Co1—P2—O1B	53.52 (14)	P2—Co1—C32—C36	158.71 (19)
P1—Co1—P2—O1B	−159.94 (9)	C35—Co1—C32—C33	81.3 (3)
P3—Co1—P2—O1B	−69.81 (9)	C34—Co1—C32—C33	37.1 (2)
C35—Co1—P2—O2B	−63.1 (4)	C36—Co1—C32—C33	119.1 (3)
C34—Co1—P2—O2B	−84.57 (15)	P1—Co1—C32—C33	−174.3 (2)
C32—Co1—P2—O2B	−17.27 (13)	P3—Co1—C32—C33	35.3 (4)
C36—Co1—P2—O2B	1.64 (17)	P2—Co1—C32—C33	−82.2 (2)
C33—Co1—P2—O2B	−56.21 (13)	C36—C32—C33—C34	0.3 (3)
P1—Co1—P2—O2B	90.33 (9)	Co1—C32—C33—C34	−60.2 (2)
P3—Co1—P2—O2B	−179.54 (9)	C36—C32—C33—Co1	60.5 (2)
C35—Co1—P3—O3P	172.13 (18)	C35—Co1—C33—C34	38.0 (2)
C34—Co1—P3—O3P	−147.70 (17)	C32—Co1—C33—C34	118.7 (3)
C32—Co1—P3—O3P	−146.0 (3)	C36—Co1—C33—C34	81.6 (3)
C36—Co1—P3—O3P	151.80 (16)	P1—Co1—C33—C34	128.5 (2)
C33—Co1—P3—O3P	−120.05 (16)	P3—Co1—C33—C34	−47.6 (3)
P1—Co1—P3—O3P	62.11 (9)	P2—Co1—C33—C34	−139.3 (2)
P2—Co1—P3—O3P	−27.02 (9)	C35—Co1—C33—C32	−80.7 (2)
C35—Co1—P3—O2C	−66.20 (18)	C34—Co1—C33—C32	−118.7 (3)
C34—Co1—P3—O2C	−26.03 (18)	C36—Co1—C33—C32	−37.1 (2)
C32—Co1—P3—O2C	−24.4 (3)	P1—Co1—C33—C32	9.8 (3)
C36—Co1—P3—O2C	−86.53 (17)	P3—Co1—C33—C32	−166.27 (17)
C33—Co1—P3—O2C	1.62 (17)	P2—Co1—C33—C32	102.1 (2)
P1—Co1—P3—O2C	−176.22 (10)	C32—C33—C34—C35	0.5 (4)
P2—Co1—P3—O2C	94.65 (10)	Co1—C33—C34—C35	−59.6 (2)
C35—Co1—P3—O1C	47.72 (18)	C32—C33—C34—Co1	60.1 (2)
C34—Co1—P3—O1C	87.88 (17)	C35—Co1—C34—C33	−119.0 (3)
C32—Co1—P3—O1C	89.6 (3)	C32—Co1—C34—C33	−37.6 (2)
C36—Co1—P3—O1C	27.39 (17)	C36—Co1—C34—C33	−80.5 (2)
C33—Co1—P3—O1C	115.54 (16)	P1—Co1—C34—C33	−120.6 (3)
P1—Co1—P3—O1C	−62.30 (9)	P3—Co1—C34—C33	141.8 (2)
P2—Co1—P3—O1C	−151.43 (9)	P2—Co1—C34—C33	49.2 (2)
O3—V1—O1—C1	−104.15 (14)	C32—Co1—C34—C35	81.4 (2)
O1P—V1—O1—C1	158.36 (13)	C36—Co1—C34—C35	38.4 (2)
O3P—V1—O1—C1	64.2 (3)	C33—Co1—C34—C35	119.0 (3)
O2—V1—O1—C1	−8.84 (12)	P1—Co1—C34—C35	−1.6 (4)
O2P—V1—O1—C1	73.79 (12)	P3—Co1—C34—C35	−99.3 (2)
O3—V1—O2—C2	103.96 (13)	P2—Co1—C34—C35	168.18 (19)
O1P—V1—O2—C2	−48.4 (3)	C33—C34—C35—C36	−1.2 (4)
O3P—V1—O2—C2	−155.87 (12)	Co1—C34—C35—C36	−61.8 (2)
O1—V1—O2—C2	5.59 (11)	C33—C34—C35—Co1	60.6 (2)
O2P—V1—O2—C2	−74.30 (12)	C34—Co1—C35—C36	117.0 (3)
V1—O1—C1—O1^i^	−169.74 (13)	C32—Co1—C35—C36	37.1 (2)
V1—O1—C1—C2	10.26 (13)	C33—Co1—C35—C36	80.2 (2)
V1—O2—C2—O2^i^	177.97 (13)	P1—Co1—C35—C36	−63.9 (2)
V1—O2—C2—C1	−2.03 (13)	P3—Co1—C35—C36	−157.4 (2)
O1—C1—C2—O2	−5.76 (13)	P2—Co1—C35—C36	87.8 (5)
O1^i^—C1—C2—O2	174.24 (13)	C32—Co1—C35—C34	−79.8 (2)
O1—C1—C2—O2^i^	174.24 (13)	C36—Co1—C35—C34	−117.0 (3)
O1^i^—C1—C2—O2^i^	−5.76 (13)	C33—Co1—C35—C34	−36.7 (2)
O2A—P1—O1P—V1	−155.10 (15)	P1—Co1—C35—C34	179.1 (2)
O1A—P1—O1P—V1	95.11 (16)	P3—Co1—C35—C34	85.7 (2)
Co1—P1—O1P—V1	−28.41 (18)	P2—Co1—C35—C34	−29.2 (5)
O3—V1—O1P—P1	153.83 (16)	C33—C32—C36—C35	−1.1 (3)
O3P—V1—O1P—P1	53.77 (16)	Co1—C32—C36—C35	59.5 (2)
O1—V1—O1P—P1	−106.88 (16)	C33—C32—C36—Co1	−60.6 (2)
O2—V1—O1P—P1	−53.9 (4)	C34—C35—C36—C32	1.4 (4)
O2P—V1—O1P—P1	−28.19 (16)	Co1—C35—C36—C32	−60.1 (2)
O1P—P1—O1A—C1A	39.5 (3)	C34—C35—C36—Co1	61.5 (2)
O2A—P1—O1A—C1A	−73.0 (3)	C35—Co1—C36—C32	118.8 (3)
Co1—P1—O1A—C1A	168.8 (2)	C34—Co1—C36—C32	79.9 (2)
O1P—P1—O2A—C3A	8.5 (3)	C33—Co1—C36—C32	37.3 (2)
O1A—P1—O2A—C3A	123.3 (2)	P1—Co1—C36—C32	−118.93 (19)
Co1—P1—O2A—C3A	−121.6 (2)	P3—Co1—C36—C32	151.20 (16)
P1—O1A—C1A—C2A	−148.7 (4)	P2—Co1—C36—C32	−30.5 (3)
P1—O2A—C3A—C4A	141.6 (3)	C34—Co1—C36—C35	−38.9 (3)
O1B—P2—O2P—V1	101.82 (15)	C32—Co1—C36—C35	−118.8 (3)
O2B—P2—O2P—V1	−148.35 (13)	C33—Co1—C36—C35	−81.4 (3)
Co1—P2—O2P—V1	−25.91 (15)	P1—Co1—C36—C35	122.3 (2)
O3—V1—O2P—P2	−164.0 (17)	P3—Co1—C36—C35	32.4 (3)
O1P—V1—O2P—P2	60.05 (13)	P2—Co1—C36—C35	−149.2 (2)

Symmetry codes: (i) −*x*, *y*, −*z*+1/2.
